# Artificial reservoirs complement natural ponds to improve pondscape resilience in conservation corridors in a biodiversity hotspot

**DOI:** 10.1371/journal.pone.0204148

**Published:** 2018-09-20

**Authors:** Charl Deacon, Michael John Samways, James Stephen Pryke

**Affiliations:** Department of Conservation Ecology and Entomology, Stellenbosch University, Matieland, South Africa; University of Sydney, AUSTRALIA

## Abstract

Natural ponds are rich in biodiversity, contributing greatly to regional aquatic biodiversity. Artificial reservoirs used for irrigation can be significant additional features of the landscape. They infill the local natural pondscape, and are attractors for aquatic insects. Here, we determine the extent to which artificial reservoirs represent the local natural pond biota, and how they contribute to the pondscape in conservation corridors used to mitigate the impact of plantation forestry in a global biodiversity hotspot. We did this by: 1) identifying the environmental factors, including plants, that drive dragonfly, water beetle, and water bug species richness, diversity and composition, and 2) determining the value of natural ponds vs. artificial reservoirs for maintaining the population size and expanding the area of occupancy for dragonflies, beetles and bugs in conservation corridors. While vegetation cover was central for maintaining species richness and composition of the assemblages in general, many other environmental variables are necessary to encourage the full suite of local diversity. Artificial reservoirs are attractive habitats to many species, overall increasing area of occupancy for 75% of them (ranging from 62–84% for different taxa). These reservoirs provide complementary alternative habitats to natural ponds, leading to improved ecological resilience across the pondscape. We conclude that maintaining a diverse and heterogeneous pondscape is important for conserving local aquatic insect diversity, and that artificial reservoirs increase the local area of occupancy for a range of pond insects in conservation corridors, and improve the biodiversity value of these pondscapes.

## Introduction

Freshwater ponds are lentic water bodies <2 ha in size [1], common throughout the world [2]. They contribute greatly to local ecology [3] and support high biodiversity, sometimes greater than that in larger water bodies such as rivers and lakes [4], due partly to their high habitat heterogeneity at the landscape scale [4]. In transformed areas with limited numbers of natural ponds, artificial reservoirs can also provide refuge habitat for rare and threatened species [5].

Artificial reservoirs are constructed for water storage [6] and may replace small, natural wetlands and ponds, especially in agricultural and urban landscapes [7; 8]. Compared to natural ponds, reservoirs are often of recent origin. Yet, reservoirs can support considerable freshwater biodiversity [6; 9], and like natural ponds, can expand the area of occupancy for many aquatic species, often supporting rare species not in the immediate area [7; 9]. This may come about through provision of essential physical characteristics, such as vegetation structure, substrate composition, and reservoir size [11; 12], or physico-chemical characteristics such as elevation, temperature, and pH [8].

Groups of ponds, natural and/or artificial, across a landscape are known as pondscapes [3]. They are important in conservation efforts, as they support higher community diversity than single large ponds or reservoirs [11; 13]. Pondscapes have been poorly explored in areas of the world with exceptionally high biodiversity. One of these areas is the Maputaland-Pondoland-Albany (MPA) biodiversity hotspot in South Africa, where large-scale conservation corridors of remnant land are in place to mitigate timber production [14]. These conservation corridors extend across the landscape to make up large-scale ecological networks [15]. They have a rich toposcape of hills, wetlands, natural ponds, and artificial reservoirs [16].

With increasing pressures on water resources, there has been much interest in aquatic insects occupying freshwater habitats [17; 18; 19], as a wide range of aquatic habitats lend themselves to understanding landscape ecology and contribute to conservation planning [20]. Aquatic insects make up much of the total freshwater fauna [21], fulfill many ecological roles [17; 22], and have the potential to reflect the physical and biological state of ecosystems [23; 24; 25; 26]. The aquatic insects that occupy ponds, natural and artificial, in the MPA biodiversity hotspot are poorly studied, yet their diversity is likely to be high in view of what is known for dragonflies in the area [27].

Dragonflies are excellent model organisms for ecology [28; 29], as they are taxonomically well-known, adults are easy to identify in field, they are highly mobile as adults, and they occupy almost any aquatic habitat. Furthermore, they are variously sensitive to environmental differences in relation to physical structure of the aquatic and aerial biotopes [30] as well as in-water physico-chemical conditions [31], leading to them being used in freshwater condition assessment, including in the MPA hotspot [29].

Two additional insect groups receiving increasing attention as indicators of water quality are aquatic beetles and bugs [32; 33; 34]. Twenty-four families of strictly aquatic beetles [35] and 17 families of true bugs [36] are known from South Africa. Aquatic beetles and bugs are highly mobile as adults, possess several unique morphological characteristics, have adaptations to various ecological conditions [37; 38], and fulfill many roles in many aquatic ecosystems [35; 39]. At the family level, water beetles and water bugs variously respond to physico-chemical change and vegetation structure [33; 39; 40]. However, especially in South Africa, their taxonomy and distribution are not well known, restricting their use as bioindicators.

Little research has been undertaken using a range of aquatic taxa for assessing natural vs. artificial ponds, especially in conservation corridors designed and managed principally using terrestrial taxa and interactions. One study considers the drivers of the composition of various aquatic insect taxa composition in the MPA hotspot [41], in addition to that of dragonflies [e.g. 40; 42; 43].

Here, we focus on the value of pondscapes as conservation clusters, and: 1) identify the physical and environmental variables driving dragonfly, water beetle, and water bug species richness, diversity, and composition in the MPA hotspot, and 2) determine the ecological value of artificial reservoirs vs. natural ponds for maintaining population sizes and expanding the local area of occupancy for dragonflies, beetles and bugs in conservation corridors. As conservation corridors have proven to be an effective conservation measure for terrestrial and aquatic ecosystems, we identify the important features of artificial reservoirs relative to natural ponds for maintaining aquatic insect diversity across this production landscape.

## Sites, materials and methods

### Study sites

**Fig 1:**
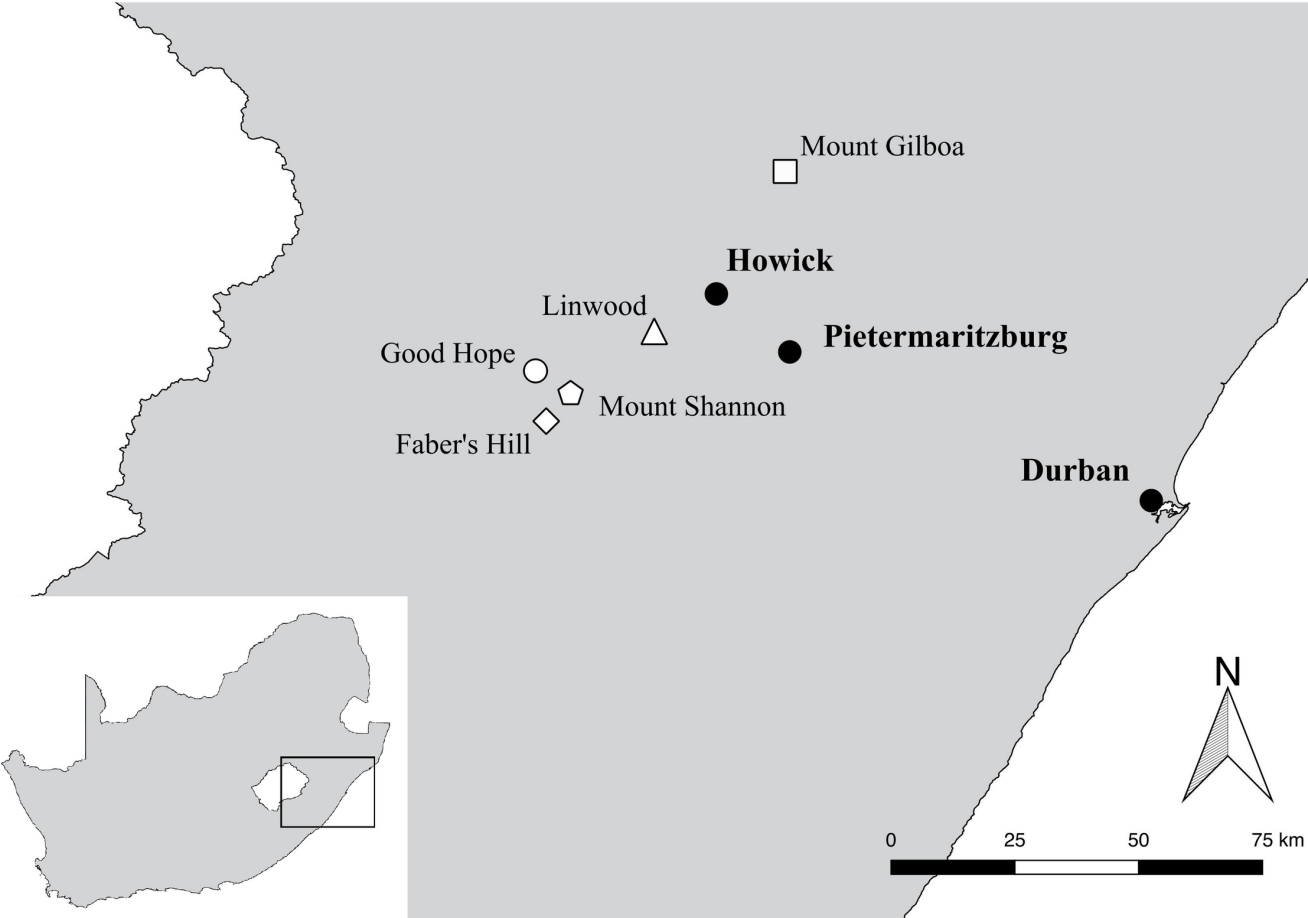
Locations of sampling areas in the Maputaland-Pondoland-Albany biodiversity hotspot. Pentagon: Mount Shannon Estate, diamond: Faber’s Hill Estate, circle: Good Hope Estate, triangle: Linwood Estate, and square: Mount Gilboa Estate. Black circles represent nearby towns.

Forty study sites were selected in the KwaZulu-Natal Midlands, South Africa: 20 natural ponds and 20 artificial reservoirs, in five geographical areas (Fig 1; Table 1). Sampling sites were selected to represent a spectrum of variation in natural quality, based on past records (obtained from satellite imagery) and initial inspection. Demarcation of natural pond sites was based on the presence/absence of hydrophilic plant species, benthic slow-flow characteristics of the water body, and the geomorphological setting. Only open grassland matrix valley bottoms and plains were considered, being the position of most ponds and reservoirs. Geomorphological data were obtained from the National Freshwater Ecosystem Priority Areas (NFEPA) database.

**Table 1:** Geographic location and pond type of sampling sites in each sampling area.

Area	Site	Latitude	Longitude
Mount Shannon	Artificial reservoir 1	29° 42' 50" S	29° 59' 34" E
Mount Shannon	Natural pond 1	29° 42' 35" S	29° 58' 33" E
Mount Shannon	Natural pond 2	29° 42' 49" S	29° 59' 36" E
Mount Shannon	Natural pond 3	29° 42' 55" E	29° 59' 25" E
Faber's Hill	Artificial reservoir 1	29° 40' 26" S	29° 54' 57" E
Faber's Hill	Artificial reservoir 2	29° 40' 37" S	29° 56' 21" E
Faber's Hill	Artificial reservoir 3	29° 40' 20" S	29° 55' 10" E
Faber's Hill	Artificial reservoir 4	29° 44' 27" S	29° 54' 56" E
Faber's Hill	Natural pond 1	29° 40' 39" S	29° 55' 07" E
Faber's Hill	Natural pond 2	29° 40' 42" S	29° 54' 58" E
Faber's Hill	Natural pond 3	29° 40' 21" S	29° 56' 10" E
Faber's Hill	Natural pond 4	29° 40' 02" S	29° 56' 09" E
Faber's Hill	Natural pond 5	29° 40' 27" S	29° 56' 05" E
Faber's Hill	Natural pond 6	29° 44' 23" S	29° 55' 02" E
Mount Gilboa	Artificial reservoir 1	29° 14' 42" S	30° 20' 35" E
Mount Gilboa	Artificial reservoir 2	29° 14' 32" S	30° 19' 45" E
Mount Gilboa	Artificial reservoir 3	29° 14' 19" S	30° 20' 02" E
Mount Gilboa	Artificial reservoir 4	29° 15' 21" S	30° 19' 02" E
Mount Gilboa	Artificial reservoir 5	29° 14' 42" S	30° 17' 01" E
Mount Gilboa	Artificial reservoir 6	29° 14' 52" S	30° 19' 49" E
Mount Gilboa	Artificial reservoir 7	29° 15' 13" S	30° 18' 45" E
Mount Gilboa	Natural pond 1	29° 14' 44" S	30° 17' 40" E
Mount Gilboa	Natural pond 2	29° 15' 04" S	30° 15' 41" E
Mount Gilboa	Natural pond 3	29° 14' 59" S	30° 15' 05" E
Mount Gilboa	Natural pond 4	29° 14' 41" S	30° 19' 46" E
Mount Gilboa	Natural pond 5	29° 15' 15" S	30° 15' 37" E
Mount Gilboa	Natural pond 6	29° 15' 03" S	30° 14' 59" E
Mount Gilboa	Natural pond 7	29° 15' 02" S	30° 15' 03" E
Mount Gilboa	Natural pond 8	29° 14' 42" S	30° 19' 48" E
Good Hope	Artificial reservoir 1	29° 39' 18" S	29° 58' 12" E
Good Hope	Artificial reservoir 2	29° 39' 09" S	29° 58' 18" E
Good Hope	Artificial reservoir 3	29° 37' 40" S	29° 59' 06" E
Good Hope	Artificial reservoir 4	29° 40' 07" S	29° 58' 26" E
Good Hope	Natural pond 1	29° 39' 13" S	29° 57' 13" E
Good Hope	Natural pond 2	29° 39' 27" S	29° 58' 28" E
Good Hope	Natural pond 3	29° 40' 08" S	29° 58' 19" E
Linwood	Artificial reservoir 1	29° 33' 38" S	30° 05' 38" E
Linwood	Artificial reservoir 2	29° 32' 59" S	30° 06' 07" E
Linwood	Artificial reservoir 3	29° 33' 54" S	30° 06' 47" E
Linwood	Artificial reservoir 4	29° 33' 38" S	30° 05' 33" E

### Data collection

Data were collected during two sampling seasons: early summer (January-February) and late summer (February-March). Adult dragonfly (Odonata), water beetle (Coleoptera), and water bug (Hemiptera) individuals were sampled on cloudless, windless days, 10h30-15h30, once during each sampling season. Each insect order was treated as a separate entity, as they differ substantially in terms of habitat requirements and traits. Ten quadrats of 4 m^2^ were selected on the edge of the water body, and swept with an aquatic net (300 mm x 300 mm; 1000 micron mesh size) for 3 min to collect beetles and bugs. Quadrats were selected to represent all features of the water body at a depth of <1.2 m, below which aquatic insect diversity in this area drops off considerably [43]. Collected individuals were identified to at least genus by making use of the Water Research Commission identification guides [39], museum collections, and expert opinion (P. Reavell, pers. Comm.). In the case of adult dragonflies, two 50 m transects were selected per site, wherein all individuals were visually recorded for 30 min. Any other, large hawking species (e.g. *Anax imperator*) that were within 5 m of transects were also recorded. Dragonfly larvae were not included, as local larval taxonomy is not sufficiently well known to species level. To confirm the identity of species, individuals were collected with an insect net and identified using relevant field guides [27; 29]. The procedure was repeated for the second sampling season. Two individuals of each species sampled are kept in a reference collection at the Stellenbosch University Entomological Museum.

At each study site, ten point measurements of physico-chemical conditions were recorded at each sampling depth on cloudless, windless days, 10h30-15h30: dissolved oxygen (mg/L), water temperature (°C), conductivity (μs), turbidity (cm visibility, using a clarity tube), and sampling depth (m, using a measuring pole). In addition, water body size (m^2^) and elevation (m a.s.l.) was recorded using Garmin eTrex 30 map data. Vegetation structure and composition was determined in five quadrats of 4 m^2^ at the edge of each water body. Within each quadrat, the percentage grass cover, percentage reed cover, percentage forb cover, average vegetation height, and dominant marginal and submerged plant species were recorded.

Geomorphological data of each study site were obtained from the NFEPA database, and each site was ground-truthed. In the event of inaccurate classification in the NFEPA database due to coarse spatial scale, the particular site was reclassified in field by making use of geomorphological classification guidelines [44].

### Statistical analyses

In order to determine whether sampling was sufficient and that the subset of data was representative of the sampling area, two species estimators were calculated and compared with the number of observed species (Sobs):

$$S_{Chao2}=S_{obs}+\frac{Q_{1}^{2}}{{2Q}_{2}}$$ Chao2

Where Q1 is species occurring exclusively in one sample and Q2 species occurring in two samples and,

$$S_{jack2}=S_{obs}+[\frac{Q_{1}(2m-3)}{m}-\frac{Q_{2}{\left(m-2\right)}^{2}}{m(m-1)}$$ Jackknife2

Where m is the total number of samples collected.

The Chao2 species estimator is proven to be very effective for insect studies as non-parametric estimators are better for datasets with a large number of rare species. Jackknife2 species estimator is another effective non-parametric estimator, that is particularly unresponsive to sampling bias.

Generalized linear mixed modeling was used to pre-select and test the random and fixed effects of environmental variables on overall species richness and abundance, as well as within natural ponds and artificial reservoirs, using the *lme4* package in R [45; 46]. For species richness, the three separate models (overall effect, within natural ponds and within artificial reservoirs) were built with pond size, elevation, site type, geomorphological class of pond, vegetation height, percentage total cover, percentage reed cover, percentage forb cover, percentage grass cover, water depth, dissolved oxygen, water temperature, water conductivity, water pH, and turbidity as fixed variables, and the sampling season and area where the sites were located as random variables. For species abundance, a single overall model was built with pond type as fixed variable, and area where site is located, sampling season and site identity as random variables, to determine the difference in abundance between natural ponds and artificial reservoirs. All generalized linear mixed models were fitted by a Laplace approximation and a Poisson distribution. For all significant regressions, we used piece-wise regressions to segregate environmental data and determine the breakpoint in each regression using the *segmented* package in R [46; 47].

The Shannon diversity index (hereafter referred to as “diversity”), accounting for species abundance and evenness, was calculated for each insect order at each sampling site in R using the *vegan* package [46; 48] and log-transformed. Linear mixed modeling was then used to pre-select and then test the random and fixed effect of environmental variables on overall insect diversity, as well as within natural ponds and artificial reservoirs. The three separate models (overall effect, within natural ponds and within artificial reservoirs) were again built with site size, elevation, site type, geomorphological class of site, vegetation height, percentage total cover, percentage reed cover, percentage forb cover, percentage grass cover, water depth, dissolved oxygen, water temperature, water conductivity, water pH, and turbidity as fixed variables, and the sampling season and area where the sites were located as random variables. The linear models were fitted by a Laplace approximation and a normal distribution. In the case of categorical fixed variables, categorical pairwise t-tests and Tukey post-hoc tests were used to determine significance. Again, for all significant regressions, we used piece-wise regressions to segregate environmental data and determine the breakpoint in each regression using the *segmented* package in R [46; 47].

Distance-based linear modeling (DistLM), based on resemblance matrices and effects of multiple predictor variables, was performed to explain the variation in species composition using recorded environmental variables, in PRIMER version 6 [49]. Forward selection of environmental variables was used, meaning that each environmental variable was added into the analysis until no significant effect on the species composition was evident. In addition, permutational multivariate analyses of variance (PERMANOVA) were used to determine the difference in environmental variables between natural ponds and artificial reservoirs. 9999 permutations were used to determine effects of environmental variables on the overall species composition of the three orders, as well as within each water body type. Permutational analyses were used to randomize factors and to select the factors that explained species composition the best. The Bray-Curtis similarity measure (which measures species composition based on the abundance of each species) was used to evaluate species composition of all groups.

## Results

A total of 61 lentic species were sampled (4 895 individuals), comprising 27 dragonfly species (1 053 individuals), 16 beetle species (658 individuals) and 18 bug species (3 184 individuals). The number of observed species (Sobs) neared the estimated number of species (Chao2 and Jackknife2) across the insect orders, as well as within each pond type. This indicated that sampling was sufficient, and that the subset of data is representative (S1 Table). For the complete list of species, refer to S2 Table.

### Influence of environmental variables on species richness, abundance and insect diversity

Overall dragonfly species richness and diversity increased with an increase in water temperature, but diversity decreased with an increase in % forb cover (Table 2). There was a significant negative relationship between water body size, and dragonfly species richness, although dragonfly diversity increased with water body size, until a size of about 20 260 m^2^ was reached, after which diversity decreased significantly (t = 10.4; p < 0.001). There was no significant difference between dragonfly abundance for natural ponds and artificial reservoirs (t = 0.6; p = 0.532). For natural ponds, dragonfly species richness and diversity increased with an increase in water temperature and % reed cover. Natural ponds in valley bottoms had significantly higher dragonfly species richness and diversity over natural ponds on open plains. In the case of artificial reservoirs, dragonfly species richness decreased with an increase in water body size. An increase in dissolved oxygen, sampling depth, % reed cover, and % grass cover gave an increase in dragonfly species richness. Dragonfly diversity decreased with an increase in dissolved oxygen, and in the case of % forb cover, decreased until about 22% cover was reached, above which there was an increase in diversity (t = 9.8: p < 0.001). Dragonfly diversity increased with water body size until about 15 400 m^2^, after which diversity decreased (t = 11.5; p < 0.001). For a summary on the ranges of measured environmental variables, consult S3 Table.

**Table 2:** Effects of environmental variables on the overall species richness and diversity, and in the two water body types, natural vs. artificial.

		Overall		Natural Ponds		Artificial reservoirs	
		Species richness	Shannon index	Species richness	Shannon index	Species richness	Shannon index
**Dragonflies**	**Water body size**	(-)4.091*	(+/-)13.036***			(-)17.066***	(+/-)26.098***
** **	**Temperature**	(+)8.815**	(+)10.584**	(+)6.521*			
** **	**Dissolved oxygen**					(+)10.919***	(-)4.196*
** **	**Depth**					(+)6.64**	
** **	**% Reed cover**			(+)7.191*	(+)3.894*	(+)4.660*	
** **	**% Grass cover**					(+)3.928*	
** **	**% Forbs cover**		(-)4.104*				(-/+)5.284*
** **	**Geomorph class**		-3.154**	-2.26*	-6.252***		
**Beetles**	**Depth**	(-)9.376**		(-)4.403*			
** **	**Temperature**	(-)4.285*	(-)4.408*	(+)8.523**	(+)23.155***		
** **	**Conductivity**	(-)4.436*	(-)4.743*				
** **	**pH**				(+)15.795***		
** **	**Dissolved oxygen**				(-)17.851***		
** **	**Elevation**				(-/+)8.317**		
** **	**% Forbs cover**					(+)9.526**	(+)5.351*
** **	**% Reed cover**				(-)9.970**		
** **	**Water body type**	3.07**	2.636*				
**Bugs**	**Temperature**	(+)5.564*		(+)7.361*	(+)4.328*		
** **	**Conductivity**	(-)6.743*					
** **	**% Grass cover**					(-/+)9.814**	

Chi square values are indicated, and t-values are indicated in bold in the case of categorical variables. (+): positive correlation; (-): negative correlation; (+/-): initial positive correlation; (-/+): initial negative correlation.

Significance levels

*: p < 0.05

**: p < 0.01

***: p < 0.001.

Type of water body (natural pond vs. artificial reservoir) had a significant effect on overall beetle species richness (Table 2), with natural ponds supporting more species. Overall beetle species richness increased with a decrease in water temperature and conductivity, while species richness decreased with an increase in water depth. There was no significant difference between beetle abundance for natural ponds and artificial reservoirs (t = -0.3; p = 0.077). For natural ponds, beetle species richness and diversity increased with water temperature, although beetle species richness decreased with increasing water depth. Beetle diversity increased with an increase in water pH, and decreased with increased dissolved oxygen and % reed cover. Beetle diversity decreased with increasing elevation, but above 1 500 m a.s.l., diversity increased (t = 0.9; p = 0.036). For artificial reservoirs, beetle species richness and diversity increased with increased % forb cover.

Overall bug species richness increased with water temperature but decreased with increased conductivity (Table 2). There was no significant difference between bug abundances for natural ponds and artificial reservoirs (t = 0.1; p = 0.928). For natural ponds, bug species richness and diversity increased with water temperature. For artificial reservoirs, bug species richness decreased with an initial increase % grass cover but increased above 13% grass cover (t = 9.795; p < 0.001).

### Influence of environmental factors on dragonfly, beetle and bug assemblages

**Fig 2:**
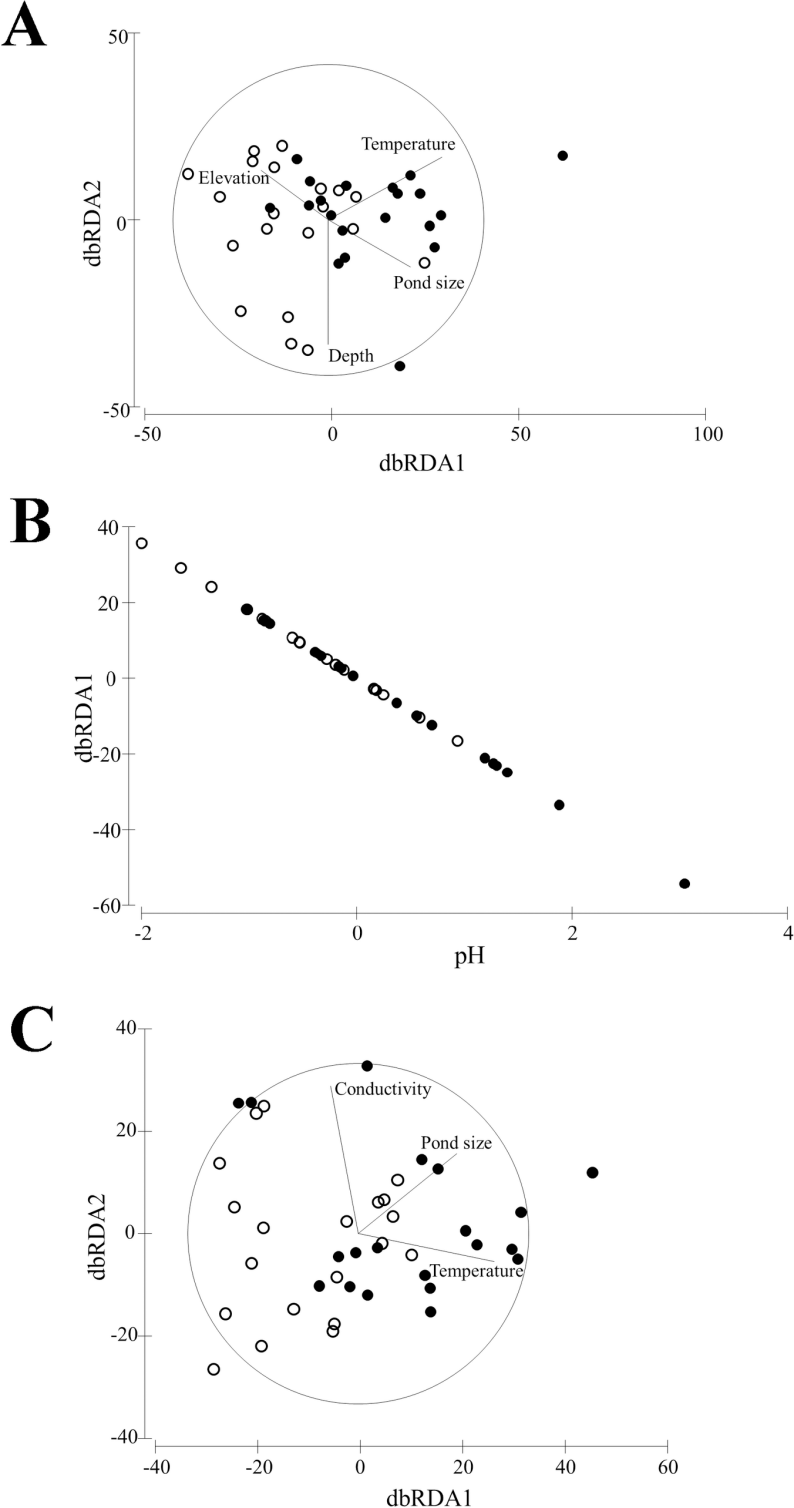
Distance-based redundancy analysis (dbRDA) results indicating significant effects of environmental variables on insect species composition. Vectors represent the effect of environmental variables on dragonfly (A), beetle (B) and bug (C) species composition between natural ponds (open circles) and artificial reservoirs (filled circles). Axes represent Bray-Curtis distance measure.

Pond type (natural vs. artificial) had a significant effect on dragonfly (pseudo-F = 3.08), beetle (pseudo-F = 3.12) and bug (pseudo-F = 2.97) assemblages, respectively. Within each water body type, the geomorphological class had no effect on the species assemblage of any of the three groups. Of the 13 environmental variables measured, distance based on linear modeling (DistLM) selected six variables as significant to overall aquatic insect species composition. These were water temperature, pH, conductivity, depth, pond size and elevation (Table 3; Fig 2). Water turbidity, dissolved oxygen, % reed cover, % forb cover, total % vegetation cover, and vegetation height did not influence species composition. For overall dragonfly species composition, 14.29% of the variation was explained by water temperature, 7.83% explained by water body size, 4.79% explained by elevation, and 1.93% explained by water depth.

**Table 3:** Distance based on linear modeling (DistLM) sequential results indicating environmental variables most descriptive of aquatic insect species composition structure between habitat types.

Group	Type	Environmental variables	F	Variation explained (%)	Cumulative variation explained (%)
**Dragonflies**	**Overall**	Temperature	5.181***	14.29	14.29
		Water body size	2.792**	7.83	22.12
		Elevation	2.437*	4.79	26.91
		Depth	2.692*	1.93	28.84
	**Ponds**	Temperature	4.556***	20.2	20.2
	**Reservoirs**	Temperature	3.177**	19.01	19.01
** **	** **	Water body size	3.033**	27.87	46.88
**Beetles**	**Overall**	pH	3.522***	8.48	8.48
	**Ponds**	pH	2.288*	11.28	11.28
** **	**Reservoirs**	Depth	1.748*	8.85	8.85
**Bugs**	**Overall**	Temperature	3.535***	11.56	11.56
		Conductivity	3.032**	6.84	18.4
		Water body size	2.308*	2.13	20.53
	**Ponds**	Temperature	3.399***	15.89	15.89
** **	**Reservoirs**	% Grass cover	4.122*	18.63	18.63

Significance levels

*: p < 0.05

**: p < 0.01

***: p < 0.001.

For overall beetle species composition, water pH explained 8.48% of total variation, and for overall bug species composition 11.56% of the variation was explained by water temperature, 6.84% by conductivity, and 2.13% by water body size. Two variables (water temperature and pH) influenced species composition in natural ponds (Table 3). Water temperature explained 20.2% of the variation in dragonfly species composition, and 15.89% of the variation in bug species composition. Water pH explained 11.28% of the variation in beetle species composition. Four environmental variables influenced species composition in artificial reservoirs. These were water temperature, depth, water body size, and % grass cover. Water temperature and water body size explained 19.01% of the variation and 27.87% of the variation in dragonfly species composition respectively. Sampling depth explained 8.85% of the variation in beetle species composition and % grass cover explained 18.63% of the variation in bug species composition.

15% of the sampled dragonfly species were unique to natural ponds (Fig 2). These were *Chlorolestes fasciatus*, *Elattoneura glauca*, *Proischnura rotundipennis* and *Zosteraeshna minuscula*. No dragonfly species was unique to the artificial reservoirs. 25% of beetle species was unique to natural ponds (Fig 2), and included *Amphiops* sp., *Aulonogyrus* sp. 1, *Copelatus* sp., *Derovatellus* sp. and *Orectogyrus* sp., and 13% unique to artificial reservoirs, and included *Aulonogyrus* sp. 2 and *Hydropeplus* sp. 28% of the bug species sampled was unique to natural ponds (Fig 2). These were *Borborophilus afzelii*, *Laccotrephes brachialis*, *Limnogonus capensis*, *Ranatra grandicollis* and *Sigara pectoralis*. No bug species were unique to the artificial reservoirs.

## Discussion

### Relative significance of the environmental variables

We found that any one of the investigated habitat descriptors cannot be substituted by another, and the focal taxa respond to each in different ways. As a result, high habitat heterogeneity created by the combination of environmental variables and pond types which maintain high insect diversity. Aquatic habitat heterogeneity in the form of vegetation complexity, substrate structure, and/or physico-chemical characteristics are important for aquatic insects [51; 53; 54], and adult, terrestrial dragonflies [31], as we found here. This supports earlier studies [19; 22; 31; 50; 51], whether at the regional scale [52], or at the finer scale of the pondscape [3; 15].

Many dragonflies occupying lentic habitats require marginal and/or submerged vegetation [19; 27; 29] as perching sites, substrate for larvae to seek refuge, and to emerge as adults [10]. Furthermore, vegetation provides habitat for food items for both adult and larval dragonflies. Here, margins of natural ponds were predominantly covered by a mixture of grasses and forbs, with little variation between ponds, and were neither extensive, nor casting much shade which otherwise diminishes local dragonfly assemblages [30].

Artificial reservoirs had less marginal grasses and forbs coverage, more reed coverage, and were rich in submerged aquatic weeds (dominated by *Elodea* spp.). These seemingly open habitats favour early aquatic beetle and bug colonization [55; 56; 57]. However, consistent with recent suggestions [19; 22; 58], beetle species richness and diversity was positively correlated with increased forb cover in artificial reservoirs. Vegetation with complex growth forms allows Dytiscidae and Gyrinidae to exit the water when macerating prey [59], allows for the completion of their life cycles [60], and provides refuge against predators [61], all of which are important for improving their persistence. As reeds are generally tall and throw much shade, few beetles select reedy stands as microhabitat. We found that only grass cover significantly drove bug species composition in artificial reservoirs, the reason for which is possibly the presences of some scavenger families (here, Hebridae, Hydrometridae and Veliidae) having a strong preference for vegetated margins, being surface dwellers that require emergent vegetation as refuge [22; 35].

Consistent with previous studies, adult dragonflies [31; 62], aquatic beetles [19; 41] and aquatic bugs [51] respond to in-water physico-chemical conditions. Although it has been suggested that adult dragonflies are likely unable to assess water biochemistry directly [26], it has been found that in South Africa they actually can do so [31], but this is secondary to the primary response to certain visual cues from vegetation [10; 63; 64].

The beetles and most bugs sampled here are water-dwelling during their adult and larval stages, and might also be capable of assessing water biochemistry directly. Nevertheless, moderate water temperature increases activity [43; 57] and shortens larval development time [10] for dragonflies, beetles and bugs occupying both natural ponds and artificial reservoirs. For these reasons, most aquatic beetles occupy ponds characterized by moderate water temperature, yet dragonflies (and their larvae) and bugs may be able to tolerate slightly elevated water temperature. The effect of dissolved oxygen on dragonflies was only detected in artificial reservoirs, where oxygen levels were much more variable. In general, dragonfly larvae are reliant on dissolved oxygen for respiration, unlike adult beetles and bugs, being atmospheric breathers [35; 65]. Artificial reservoirs rich in dissolved oxygen were predominantly occupied by zygopterans, as most anisopterans (the majority sampled here) are physiologically better equipped to tolerate low dissolved oxygen conditions. Furthermore, high aquatic beetle diversity in natural ponds was associated with lower dissolved oxygen, as higher dissolved oxygen might be synonymous with presence of predatory vertebrate species, although not directly measured here.

Fluctuating conductivity as a proxy for salinity [66] determines overall aquatic beetle and bug species richness, and shapes aquatic bug assemblages, as high salinity interferes with metabolic capabilities and water retention in aquatic insects [67]. Here, only aquatic beetle diversity in natural ponds showed a response to water pH, suggesting that most of our aquatic beetles have a strong preference for slightly alkaline waters, related to their physiology and development [68].

Overall, natural ponds and artificial reservoirs were clearly distinct in physical characteristics (size and depth). Artificial reservoirs here were much larger and deeper than the natural ponds. Island biogeography theory suggests that larger water bodies should sustain higher species richness and a more complex species composition [69]. However, our findings suggest that dragonflies have a preference for maintained intermediate-sized natural ponds, as they provide a suitable number of microhabitats throughout the season, reducing competition for resources [14; 70]. The water level of large artificial reservoirs may also fluctuate more than natural ponds as a result of agricultural abstraction and seasonal variation [14; 27]. Similarly, the water level of small natural ponds may fluctuate greatly between seasons. In both cases, marginal and submerged vegetation is exposed [70], increasing competition for suitable microhabitats, as was the case in our area [71].

We found that water body size and depth are less important to chance colonization by aquatic beetles and bugs. Most are highly mobile as adults and occupy mostly the shallow edges regardless of water body size [22; 72]. Water depth > 1.2 m sees a great drop in temperature and dissolved oxygen, combined with a decrease in aquatic insect richness and abundance in our area [43]. Although size and depth are apparently of lesser importance to them than fine vegetation characteristics, larger and often deeper artificial reservoirs are still occupied by widespread generalist species (here, the largest proportion of beetles and bugs sampled), partly because larger size of a water body means a higher likelihood of being found by aerial and potential colonizing individuals moving across the landscape [69; 73].

We found that geomorphology of natural ponds was a significant descriptor of dragonfly species richness and diversity, but not of aquatic beetle and bug species richness, diversity or composition, suggesting that most beetle and bug species here occupy both the grassy valley-bottom and open plain ponds. Geomorphology cannot fully be used as a measure of permanency, but remains important to consider as it is likely to be related to the relative age of ponds, determine variability in physico-chemical characteristics [31] and climatic factors such as wind speed, surface water run-off, and variation in marginal/submerged vegetation structure [44], all of which contribute to habitat heterogeneity.

Among the narrow range of elevations we investigated (~1100–1500 m), there was little effect of elevation on overall dragonfly species richness and diversity, but wide elevation gradients over hundreds of meters significantly influence dragonfly species assemblages in this region [42]. Here, the three dragonfly species *C*. *fasciatus*, *A*. *nigridorsum* and *P*. *jucunda* were present only at the highest elevations, *A*. *pinheyi* and *Z*. *minuscula* at intermediate elevations, and *P*. *rotundipennis* only at low elevations. With changing elevation, habitat characteristics related to vegetation composition and temperature change, result in a subsequent change in dragonfly species’ assemblages as different microhabitats become available/unavailable [42; 74]. Here, low beetle diversity was associated with natural ponds at intermediate elevations, but showed an increase at higher elevations. High beetle diversity probably arises from high species turnover between lentic and lotic habitats at higher elevations [75; 76] and high microhabitat availability in natural ponds at low elevations respectively [25]. Increased aquatic bug species richness and diversity can be associated with increased elevation [57], but we did not find that here across our short elevation range. Aquatic bugs in this area are mainly widespread generalists [35; 36] possessing great plasticity [77], allowing them to occupy a variety of aquatic environments. The range of elevations investigated here might simply have been too narrow to detect differences in bug species richness and assemblages.

### Added ecological value of artificial reservoirs

Range-restricted species (e.g. *P*. *rotundipennis*, a localized endemic damselfly), habitat-specific species (e.g. deposition pools coupled with bushes for oviposition, *C*. *fasciatus* (a damselfly); cool, shallow water, *B*. *afzelii* and *L*. *brachialis*, both bugs) and nearly one third of beetles did not occupy the artificial reservoirs, as their preferred microhabitats were only available in natural ponds. Nevertheless, most of the sampled species were shared between natural ponds and artificial reservoirs (75% overall; 84% of dragonflies, 62% of beetles and 72% of bugs), including two South African endemic dragonflies (*A*. *sapphirinum* and *A*. *leucosticta*), and one endemic beetle (*Algophilus* sp.).

Comparatively, there was little difference in abundance between natural ponds and artificial reservoirs across all three insect taxa. This suggests that artificial reservoirs function well in maintaining local population sizes, expanding the area of occupancy and, as natural ponds and artificial reservoirs are interspersed and close together, improve functional connectivity for most pond species. However equally important is the landscape context, as many species require areas away from water to mature, forage, roost, and seek out hibernation sites [78]. The conservation management activity of setting aside remnant corridors in and among plantation compartments provides suitable habitats and makes up ecological networks [15] improving ecological resilience across the pondscape for aquatic insects. Although not directly measured here, dispersal ability should in part determine how well aquatic insects use landscape-scale ecological networks in addition to ecological preference [79; 80]. Most aquatic insects sampled here are highly mobile as adults, enabling them to move readily between interspersed lentic habitats and track favorable ecological conditions [22; 78; 81; 82].

## Conclusion

The relatively similar environmental conditions in artificial reservoirs and natural ponds meant that most local species occupied the reservoirs (through similar levels of species richness, abundance and assemblage composition), emphasizing their great conservation value. Importantly however, it is the whole pondscape that is required to provide the wide range of environmental variables necessary to support this diversity. This is supported by no one environmental variable driving all the aquatic diversity in the same way. A range of pond types does this, as they provide a range of abiotic and biotic conditions. While the ideal is to achieve this with only natural ponds to support all the local diversity, reservoirs nevertheless go a long way to enhance the local abundance of most aquatic species.

Nearly a quarter of the species occupied only natural ponds, indicating the fundamental importance of natural ponds if we are to conserve all the local aquatic diversity. Yet, artificial reservoirs as part of a functioning pondscape in large-scale conservation corridors, improve much aquatic diversity and abundance, so contributing to improved resilience in the face of climate and land-use change. They do this principally by increasing the area of occupancy for most species.

## Supporting information

S1 Table Abundance, number of observed species (Sobs) and species estimators (Chao2 and Jackknife2). (DOCX)

S2 Table Species list of aquatic insect species sampled. * = South African endemic, ✓ = Occupying artificial reservoirs, ✗ = Occupying natural ponds
(DOCX)

S3 Table Summary statistics of environmental variables for artificial reservoirs and natural ponds. (DOCX)
