# Supplementary material for: Artificial reservoirs complement natural ponds to improve pondscape resilience in conservation corridors in a biodiversity hotspot
Source: PLoS One. 2018 Sep 20;13(9):e0204148. doi: 10.1371/journal.pone.0204148 (PMC6147492; doi:10.1371/journal.pone.0204148)
Supplement: S1 Table — (DOCX) [file pone.0204148.s001.docx]

**S1 Table. Abundance, number of observed species (Sobs) and species estimators (Chao2 and Jackknife2).**

| **Group** | **Type** | **Abundance** | **Sobs** | **Chao2** | **Jackknife2** |
| --- | --- | --- | --- | --- | --- |
| Dragonflies | Overall | 1129 | 27 | 26.67 (±1.31) | 27.07 |
|  | Pond | 438 | 27 | 38.25 (±13.15) | 37.24 |
|  | Reservoir | 691 | 23 | 30.00 (±11.66) | 28.54 |
| Beetles | Overall | 658 | 16 | 16.00 (±0) | 12.30 |
|  | Pond | 415 | 14 | 14.00 (±0) | 12.29 |
|  | Reservoir | 243 | 12 | 13.50 (±2.29) | 14.99 |
| Bugs | Overall | 3078 | 18 | 18.50 (±1.32) | 19.00 |
|  | Pond | 1038 | 18 | 18.67 (±1.31) | 19.14 |
|  | Reservoir | 2040 | 13 | 13.17 (±0.54) | 12.29 |
