# Supplementary material for: Artificial reservoirs complement natural ponds to improve pondscape resilience in conservation corridors in a biodiversity hotspot
Source: PLoS One. 2018 Sep 20;13(9):e0204148. doi: 10.1371/journal.pone.0204148 (PMC6147492; doi:10.1371/journal.pone.0204148)
Supplement: S2 Table — * = South African endemic, ✓ = Occupying artificial reservoirs, ✗ = Occupying natural ponds (DOCX) [file pone.0204148.s002.docx]

**S2 Table. Species list of aquatic insect species sampled.** * = South African endemic, ✓ = Occupying artificial reservoirs, ✗ = Occupying natural ponds

|  | Family | Artificial reservoir | Natural pond | Faber’s Hill | Mount Gilboa | Good Hope | Linwood | Mount Shannon |
| --- | --- | --- | --- | --- | --- | --- | --- | --- |
| **Odonata** |  |  |  |  |  |  |  |  |
| *Acisoma variegatum* | Libellulidae | ✓ | ✗ |  |  | ✓ |  | ✗ |
| *Africallagma glaucum* | Coenagrionidae | ✓ | ✗ | ✗ | ✓✗ | ✗ | ✓ | ✓✗ |
| *Africallagma sapphirinum** | Coenagrionidae | ✓ | ✗ | ✓✗ | ✓ | ✓ |  | ✓ |
| *Agriocnemis pinheyi* | Coenagrionidae | ✓ | ✗ |  | ✓ | ✗ |  |  |
| *Allocnemis leucosticta** | Platycnemididae | ✓ | ✗ | ✗ | ✓ | ✗ |  | ✗ |
| *Anax imperator* | Aeshnidae | ✓ | ✗ |  | ✓✗ | ✓✗ | ✓ | ✓ |
| *Anax speratus* | Aeshnidae | ✓ | ✗ | ✗ | ✗ |  | ✓ | ✓✗ |
| *Azuragrion nigridorsum* | Coenagrionidae | ✓ | ✗ | ✓ |  |  |  |  |
| *Chlorolestes fasciatus* | Synlestidae |  | ✗ |  | ✗ |  |  |  |
| *Crocothemis erythraea* | Libellulidae | ✓ | ✗ | ✓✗ | ✓ | ✓✗ | ✓ | ✓✗ |
| *Ellatoneura glauca* | Platycnemididae |  | ✗ | ✗ | ✗ |  |  | ✗ |
| *Ischnura senegalensis* | Coenagrionidae | ✓ | ✗ | ✓✗ | ✓ | ✓✗ | ✓ | ✗ |
| *Lestes plagiatus* | Lestidae | ✓ | ✗ | ✓✗ | ✓ | ✓✗ |  | ✓✗ |
| *Nesciothemis farinosa* | Libellulidae | ✓ | ✗ | ✓✗ | ✓ | ✓✗ | ✓ |  |
| *Notogomphus praetorius* | Gomphidae | ✓ | ✗ | ✓✗ | ✓✗ | ✓ |  | ✓ |
| *Orthetrum julia* | Libellulidae | ✓ | ✗ | ✓✗ | ✓✗ | ✓✗ | ✓ | ✗ |
| *Palpopleura jucunda* | Libellulidae | ✓ | ✗ | ✓✗ |  |  |  |  |
| *Pantala flavescens* | Libellulidae | ✓ | ✗ |  | ✓✗ | ✓✗ | ✓ | ✓✗ |
| *Paragomphus cognatus* | Gomphidae | ✓ | ✗ | ✓ | ✓✗ | ✓✗ |  |  |
| *Proischnura rotundipennis** | Coenagrionidae |  | ✗ |  |  |  |  | ✗ |
| *Pseudagrion caffrum* | Coenagrionidae | ✓ | ✗ | ✓ | ✓✗ | ✓ |  | ✗ |
| *Pseudagrion spernatum* | Coenagrionidae | ✓ | ✗ | ✗ | ✓✗ | ✗ | ✓ | ✓ |
| *Tramea limbata* | Libellulidae | ✓ | ✗ | ✓ | ✓ | ✓✗ | ✓ | ✓✗ |
| *Trithemis arteriosa* | Libellulidae | ✓ | ✗ | ✓✗ |  |  | ✓ |  |
| *Trithemis furva* | Libellulidae | ✓ | ✗ | ✓✗ | ✓✗ | ✓✗ | ✓ | ✓✗ |
| *Trithemis stictica* | Libellulidae | ✓ | ✗ | ✓✗ | ✓✗ | ✗ | ✓ | ✓ |
| *Zosteraeshna minuscula* | Aeshnidae |  | ✗ | ✗ |  | ✗ |  | ✗ |
| **Coleoptera** |  |  |  |  |  |  |  |  |
| *Algophilus* sp.* | Hydrophilidae | ✓ | ✗ | ✓ | ✓✗ | ✓✗ | ✓ | ✓✗ |
| *Amphiops* sp. | Hydrophilidae | ✓ | ✗ | ✗ |  |  |  | ✗ |
| *Aulonogyrus* sp. | Gyrinidae |  | ✗ | ✗ |  | ✗ |  |  |
| *Aulonogyrus* sp. 2 | Gyrinidae | ✓ |  |  |  |  | ✓ |  |
| *Copelatus* sp. | Dytiscidae |  | ✗ |  |  | ✗ |  | ✗ |
| *Derovatellus* sp. | Dytiscidae |  | ✗ |  | ✗ | ✗ |  | ✗ |
| *Gyrinus* sp. | Gyrinidae | ✓ | ✗ |  | ✓✗ | ✗ |  | ✓ |
| *Helochares* sp. | Hydrophilidae | ✓ | ✗ | ✗ | ✓✗ | ✗ |  | ✗ |
| *Hydropeplus* sp. | Dytiscidae | ✓ |  |  | ✓ |  |  |  |
| *Hyphydrus* sp. | Dytiscidae | ✓ | ✗ | ✗ | ✓✗ |  |  |  |
| *Hyphydrus* sp. 2 | Dytiscidae | ✓ | ✗ | ✓✗ | ✓✗ | ✓✗ |  | ✗ |
| *Hyphydrus* sp. 3 | Dytiscidae | ✓ | ✗ | ✓✗ | ✓✗ | ✗ |  |  |
| *Orectogyrus* sp. | Gyrinidae |  | ✗ |  |  | ✗ |  | ✗ |
| *Philaccolus* sp. | Dytiscidae | ✓ | ✗ | ✓ | ✗ | ✗ |  |  |
| *Philaccolus* sp. 2 | Dytiscidae | ✓ | ✗ | ✓✗ | ✓✗ |  |  | ✗ |
| *Rhantus concolorans* | Dytiscidae | ✓ | ✗ | ✓✗ | ✗ | ✗ |  | ✗ |
| **Hemiptera** |  |  |  |  |  |  |  |  |
| *Agraptocorixa* sp. | Corixidae | ✓ | ✗ | ✓✗ | ✓✗ | ✓✗ |  | ✓ |
| *Anisops varia* | Notonectidae | ✓ | ✗ | ✓✗ | ✓✗ | ✓✗ |  | ✓✗ |
| *Appasus* *grassei* | Belostomatidae | ✓ | ✗ | ✓✗ | ✓✗ | ✓✗ | ✓ | ✓✗ |
| *Borborophilus afzelii* | Nepidae |  | ✗ | ✗ |  | ✗ |  | ✗ |
| *Enithares glauca* | Notonectidae | ✓ | ✗ | ✓✗ | ✓✗ |  |  | ✓✗ |
| *Hebrus* sp. | Veliidae | ✓ | ✗ |  | ✓✗ | ✓ |  |  |
| *Hydrometra albolineata* | Hydrometridae | ✓ | ✗ | ✗ | ✓✗ |  |  |  |
| *Laccocoris* sp. | Naucoridae | ✓ | ✗ | ✓✗ | ✓✗ | ✓✗ |  | ✗ |
| *Laccotrephes brachialis* | Nepidae |  | ✗ | ✗ |  | ✗ |  | ✗ |
| *Limnogonus capensis* | Gerridae |  | ✗ | ✗ |  |  |  |  |
| *Micronecta* sp. | Micronectidae | ✓ | ✗ | ✓✗ | ✓✗ | ✓✗ |  | ✓ |
| *Neogerris severance* | Gerridae | ✓ | ✗ | ✓✗ | ✓✗ | ✗ |  | ✗ |
| *Plea pullula* | Pleidae | ✓ | ✗ | ✓✗ | ✓✗ |  |  | ✓✗ |
| *Ranatra franarantsoana* | Nepidae | ✓ | ✗ |  | ✓ | ✓✗ |  | ✗ |
| *Ranatra grandicollis* | Nepidae |  | ✗ |  | ✗ |  |  |  |
| *Rhagovelia nigricans* | Veliidae | ✓ | ✗ | ✗ | ✗ |  | ✓ | ✗ |
| *Sigara pectoralis* | Corixidae |  | ✗ | ✗ |  |  |  | ✗ |
| *Sigara* sp. 2 | Corixidae | ✓ | ✗ | ✓✗ | ✓✗ | ✓✗ | ✓ | ✓✗ |
