# Supplementary material for: Artificial reservoirs complement natural ponds to improve pondscape resilience in conservation corridors in a biodiversity hotspot
Source: PLoS One. 2018 Sep 20;13(9):e0204148. doi: 10.1371/journal.pone.0204148 (PMC6147492; doi:10.1371/journal.pone.0204148)
Supplement: S3 Table — (DOCX) [file pone.0204148.s003.docx]

|  |  |  |  |  |
| --- | --- | --- | --- | --- |
|  | Mean | SE | Min. | Max. |
| **Artificial reservoirs** |  |  |  |  |
| Size (m^2^) | 23933.7 | 7703.8 | 871.3 | 151604.6 |
| Elevation (m a.s.l.) | 1346.1 | 39.9 | 951.0 | 1584.0 |
| Vegetation height (m) | 0.8 | 0.1 | 0.2 | 2.5 |
| Total cover (%) | 65.4 | 6.5 | 18.0 | 100.0 |
| Reeds cover (%) | 14.0 | 4.8 | 0 | 90.0 |
| Forbs cover (%) | 38.9 | 8.2 | 0 | 100.0 |
| Grasses cover (%) | 12.8 | 3.7 | 0 | 60.0 |
| Depth (m) | 0.6 | 0.1 | 0.4 | 0.9 |
| Dissolved oxygen (mg/L) | 7.4 | 0.4 | 4.8 | 11.7 |
| Temperature (°C) | 24.8 | 0.8 | 20.7 | 37.5 |
| Conductivity (ms) | 61.6 | 5.9 | 31.7 | 119.0 |
| pH | 7.4 | 0.1 | 6.8 | 8.6 |
| Turbidity (cm visibility) | 57.0 | 6.0 | 5.0 | 100.0 |
| **Natural ponds** |  |  |  |  |
| Size (m^2^) | 7955.3 | 1925.7 | 213.5 | 30832.7 |
| Elevation (m a.s.l.) | 1435.3 | 33.1 | 950 | 1550.0 |
| Vegetation height (m) | 0.8 | 0.1 | 0.04 | 1.7 |
| Total cover (%) | 83.4 | 5.5 | 16.0 | 100.0 |
| Reeds cover (%) | 2.2 | 2.2 | 0.0 | 44.0 |
| Forbs cover (%) | 38.0 | 8.4 | 0.0 | 100.0 |
| Grasses cover (%) | 43.2 | 8.9 | 0.0 | 100.0 |
| Depth (m) | 0.5 | 0.1 | 0.1 | 1.2 |
| Dissolved oxygen (mg/L) | 6.2 | 0.4 | 2.8 | 8.8 |
| Temperature (°C) | 21.5 | 0.4 | 18.0 | 24.5 |
| Conductivity (ms) | 51.1 | 3.1 | 31.9 | 81.3 |
| pH | 7.1 | 0.1 | 6.4 | 7.6 |
| Turbidity (cm visibility) | 28.2 | 4.0 | 5.0 | 70.5 |
|  |  |  |  |  |

**S3 Table. Summary statistics of environmental variables for artificial reservoirs and natural ponds.**
